# Dupilumab for treatment of primary cutaneous amyloidosis in adults: two case reports and literature review

**DOI:** 10.3389/fmed.2025.1485079

**Published:** 2025-02-05

**Authors:** Feiying Guo, Huajie Zhong, Yuan Wu, Xue Xu, Jiarong Tan, Qiang Zhou, Shunli Tang

**Affiliations:** ^1^Department of Dermatology, Huzhou Central Hospital, Fifth School of Clinical Zhejiang Chinese Medical University, Huzhou, China; ^2^Department of Dermatology, Huzhou Central Hospital, Affiliated Central Hospital of Huzhou University, Huzhou, China; ^3^Department of Dermatology, Sir Run Run Shaw Hospital, Zhejiang University School of Medicine, Hangzhou, China; ^4^Department of Otolaryngology, Huzhou Central Hospital, Affiliated Central Hospital of Huzhou University, Huzhou, China

**Keywords:** dupliumab, amyloidosis, amyloidosis–therapy, case report, review

## Abstract

Lichenoid amyloidosis (LA), a subtype of primary cutaneous amyloidosis (PCA), is featured by intensely pruritic, hyperkeratotic papules which lacks standardized treatment. Dupilumab, a human monoclonal antibody targeting for interleukin (IL)-4/13 receptor *α* chain, is widely applied in type 2 inflammation diseases treatment. This article reported two cases of refractory LA successfully treated with dupilumab and reviewed publications reporting dupilumab treatment for PCA.

## Introduction

Lichenoid amyloidosis (LA), a subtype of primary cutaneous amyloidosis (PCA), is characterized by intensely pruritic, hyperkeratotic papules typically distributed on the extensor surfaces, i.e., calves, back, forearm, and thigh (1, 2). Traditional treatments, including topical corticosteroids, tarcolimus, vitamin D3 analogues, oral anti-histamines, retinoides, cyclosporine, phototherapy, do not work well (3). Therefore, new therapeutic options are urgently needed.

Dupilumab, a human monoclonal antibody targeting for interleukin (IL)-4/13 receptor *α* chain, is widely applied in type 2 inflammation diseases treatment, i.e., atopic dermatitis (AD), nodular prurigo, and asthma (4, 5). Herein, we reported two cases of LA treated with dupilumab due to poor response to conventional treatments and achieved satisfactory response.

## Case presentation

### Case 1

**Figure 1:**
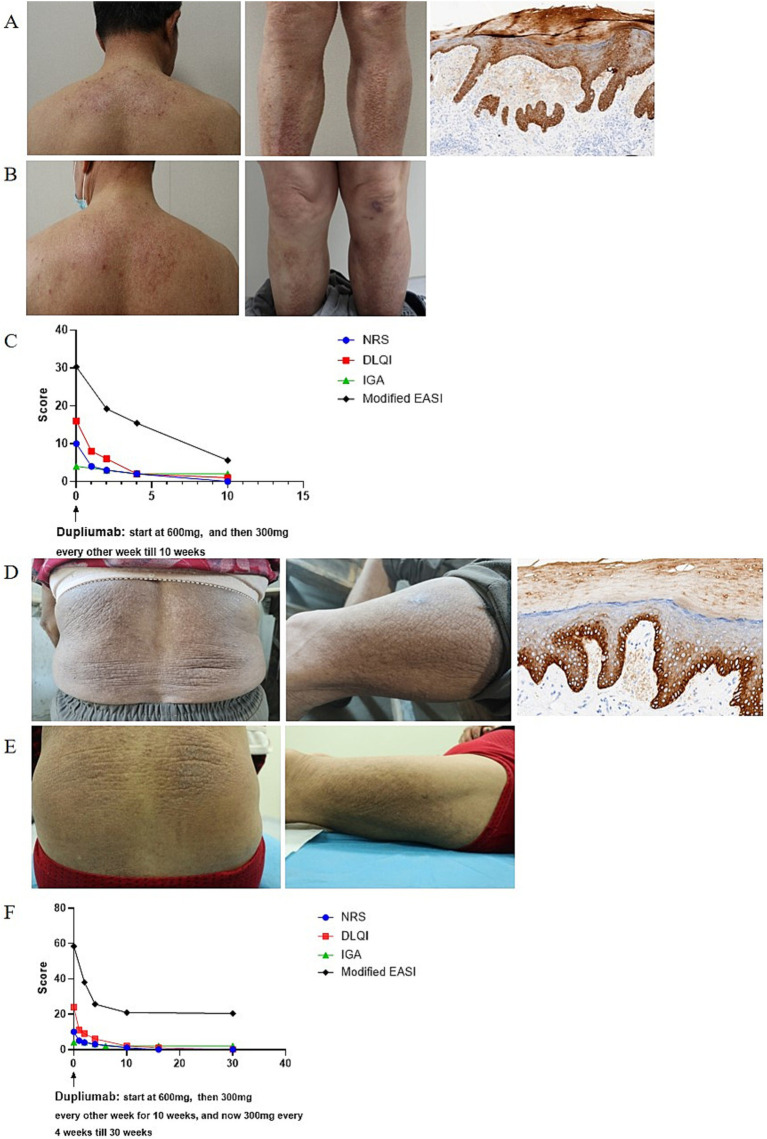
**(A,D)** Clinical and histopathological photographs at initial presentation. **(B,E)** Clinical photographs at last visit in July 2024. **(C,F)** Dupilumab treatment significantly alleviated skin lesions and pruritus. m-EASI is identical to EASI but included an additional assessment of pruritus (6).

A 66-year-old man presented to dermatology clinic with a 30-year history of generalized rashes with severe itching [Pruritus Numeric Scale Score (NRS): 10; Investigator Global Assessment (IGA): 4; Dermatology Life Quality Index (DLQI): 16, Modified Eczema area and severity index (m-EASI) (6): 30.3]. Physical examination revealed red papules, patches, and plaques symmetrically distributed on the trunk and limbs (Figure 1A). He was otherwise healthy and denied family history of similar symptoms. Laboratory examinations were unremarkable, except slightly elevated IgE levels (Serum IgE: 97 IU/mL, reference range < 87 IU/mL). A skin biopsy taken from the leg showed hyperkeratosis, dyskeratosis, acanthosis, and eosinophilic material deposition on the dermal papilla, which was positive for Congo red and CK5/6 staining (Figure 1A). Accordingly, he was diagnosed with LA. After the failure of phototherapy, topical corticosteroids, and oral anti-histamines, the patient was started with dupilumab on April 21th, 2024 at a dosage of 600 mg for first injection and 300 mg every 2 weeks thereafter. The pruritus was relieved after 1 week and the skin lesions began to improve at 2 weeks and significant subsided at 10 weeks (Figure 1B). During the treatment, all scores continued to decline (NRS:0; IGA:2; DLQI: 1; m-EASI: 5.6 at 10 weeks) (Figure 1C), without adverse reactions.

### Case 2

A 69-year-old women presented to dermatology clinic with a 3-year history of generalized rashes with severe itching (NRS: 10; IGA: 4; DLQI: 24; m-EASI: 58.4). Physical examination revealed red papules, patches, and plaques distributed on the trunk and limbs (Figure 1D). Her personal and family history were unremarkable. Laboratory examinations were all within normal range. A skin biopsy taken from the leg showed hyperkeratosis, dyskeratosis, acanthosis, and eosinophilic material deposition on the dermal papilla; eosinophilic deposition immunohistochemistry was positive for CK5/6 (Figure 1D), supporting a diagnosis of LA. After the failure of phototherapy, topical corticosteroids, and oral anti-histamines, a course of dupilumab therapy was initiated on December 27th, 2023, which started with 600 mg as a loading dose followed by 300 mg biweekly injections for 10 weeks and then 300 mg monthly injections thereafter. The pruritus was relieved after 1 week and the skin lesions improved after 2 weeks (Figure 1E). During the treatment, all scores continued to decline (NRS: 0; IGA: 2; DLQI:0; m-EASI: 20.4 at 30 weeks) (Figure 1F), without adverse reactions.

## Discussion

PCA is a frequently encountered skin disease characterized by extracellular deposition of heterogenic amyloid protein in previous normal skin in the absence of visceral organs involvement (1). Traditionally, it can be divided into macular amyloidosis, LA, and nodular amyloidosis (7). LA is the most common form of PCA, typically presenting as multiple localized or rarely generalized, hyper-pigmented grouped papules with a predilection for the calves, shins, ankles, and thighs. These lesions not only bring cosmetic concerns to patients, but also negatively impair patients’ quality of life since they are mostly associated with severe pruritus (1, 8). PCA lesions are currently considered difficult to treat, since no consistently effective therapy has been reported despite many therapeutic modalities have been tried in PCA treatment (3).

Dupilumab, a fully human anti-IL-4 receptor-*α* monoclonal antibody, blocks IL-4 and IL-13 signaling to downregulate itching-associated cytokines, chemokines, and IgE levels, which has been applied in allergic diseases treatment, i.e., AD and asthma treatment (4, 9, 10). Studies have shown that serum and cutaneous levels of type 2 cytokines (IL-4, IL-13, IL-31) and their receptors were elevated in patients with PCA, and their expression were decreased when symptoms were alleviated, indicating that type 2 inflammation may involve in LA pathogenesis (11, 12). Therefore, we speculated that dupilumab might be an alternative treatment for LA.

In this article, we successfully treated 2 refractory LA patients with dupilumab and achieved satisfactory response. The itching was relieved within 1 week after dupilumab treatment and rashes improved since 2 weeks. During the treatment, no adverse events were reported. Due to economic reasons, our patient 2 received monthly injection of dupilumab since 10 weeks after treatment onset. Though rashes showed no sustained clinical improvement since then, pruritus score and quality-of-life score maintained improvement, making the patients satisfied with therapy. We also searched the published literature reporting dupilumab treatment for PCA and summarized them in Table 1.

**Table 1:** Publications of patients with primary cutaneous amyloidosis treated with dupilumab.

Reference	Age	Gender	Disease duration	Atopic history	Other comorbidity	Blood tests	Treatment before dupilumab	Response to therapy	Adverse events
Humeda et al. (13)	76	Male	4 years	No	Prostate cancer	Serum IgE 200 kU/L Eosinophil 1.5 × 10^9^/L	UVB phototherapy Oral anti-puruitics, i.e., amitriptyline, hydroxyzine, doxepin Oral acitretin Benralizumab	Pruritus disappeared within 2 weeks, skin lesions flattened and lightened within 3 months	NA
Aoki et al. (14)	49	Female	NA	Atopic dermatitis	NA	NA	Topical corticosteroids Oral antihistamine	Pruritus decreased within 4 weeks, skin lesions improved within 3 months	No
Zahid et al. (15)	28	Female	NA	Atopic dermatitis	NA	NA	Phototherapy Topical corticosteroids Topical tacrolimus Oral cyclosporine	Pruritus improved within 3 months, skin lesions completely resolved at 6 months	No
	30	Female	NA	Atopic dermatitis	NA	NA	NA	Pruritus improved within 3 months, skin lesions completely resolved at 5 months	No
Beck et al. (16)	69	Male	NA	Asthma Allergic rhinitis	Immunoglobulin G4-related disease Chronic kinder disease (stage IV)	IgE 281 kU/L Eosinophil 2.82 × 10^9^/L ANA 1:80	Topical dapsone Topical and oral corticosteroids Oral anti-puruitics, i.e., famotidine Oral hydroxyzine Rituximab	Pruritus and skin lesions initially worsened after 1 month. Pruritus and skin lesions improved and controlled after 2 and 4 months, respectively.	NA
Zhao et al. (17)	70	Male	8 years	Atopic dermatitis	NA	Eosinophil 1.96 × 10^9^/L	Topical corticosteroids Traditional Chinese medicine	Pruritus decreased after 1 week, skin lesions improved after 4 weeks and largely subsided after 28 weeks.	No
	30	Female	25 years	Allergic rhinitis Atopic dermatitis	NA	Allergen-specific IgE: Artemisia >100 kU/L, ragweed 9.4 kU/L	Topical corticosteroids Oral antihistamines	Pruritus decreased after 1 week, skin lesions improved after 2 weeks and completely subsided after 16 weeks.	No
Zhu et al. (18)	37	Male	14 years	Allergic rhinitis Atopic dermatitis	NA	Total IgE 277.3 kU/L Eosinophil 6.30 × 10^9^/L	Topical corticosteroids Oral antihistamines	Pruritus and skin lesions improved within 16 weeks.	No
	67	Male	10 years	Allergic rhinitis Atopic dermatitis	NA	Total IgE 54.2 kU/L Eosinophil 0.10 × 10^9^/L	UVB phototherapy Topical corticosteroids Oral antihistamines	Pruritus disappeared and skin lesions improved within 16 weeks.	No
	20	Male	6 years	Allergic rhinitis Atopic dermatitis	NA	Total IgE 1002.0 kU/L Eosinophil 0.20 × 10^9^/L	Topical corticosteroids Oral antihistamines	Pruritus and skin lesions improved within 16 weeks.	NA
	54	Female	5 years	Atopic dermatitis	NA	Total IgE 893.60 kU/L Eosinophil 2.0 × 10^9^/L	Oral corticosteroids Oral antihistamines	Pruritus disappeared and skin lesions improved within 16 weeks.	No
Tirone et al. (19)	52	Female	27 years	Atopic dermatitis	NA	IgE, eosinophils increase Mites, cypress sensitiztion	Topical corticosteroids Oral cyclosporine	Pruritus and skin lesions improved within in 3 months, and completely disappeared after 1 year	No

As of October 2024, 14 patients with PCA (including our 2 patients) tried dupilumab treatment, with female to male ratio of 7:7. These patients aged 20–76 years old, and their medical history of PCA ranged from 3–27 years. All of them resisted to traditional therapy for PCA, and achieved disease relief on dupilumab treatment. Itching usually alleviated firstly, with a reported remission time of 1–12 weeks after treatment. Skin lesions improved later, which began and largely resolved after 4 weeks and 28 weeks, respectively. 4 patient patients got complete skin lesions remission, and most patients achieved significant alleviation and improvement. Notably, unlike most reported PCA patients using dupilumab, our patients denied personal and family history of atopic diseases, indicating that dupilumab may be an appropriate off-label indication for PCA treatment. Dupilumab was also successfully tried in 1 PCA patient with prostate cancer, suggesting that tumor is not a contraindication for dupilumab. However, due to the limited experience, we propose that more observation and trial of PCA treated with dupilumab are needed to confirm its efficacy and safety.

In summary, we speculate that dupilumab may be a promising therapy for PCA, with excellent effectiveness and safety. However, further studies are necessary to clarify the complex pathogenesis of PCA and the role of dupilumab in its treatment.

## Conclusion

Dupilumab may be a promising therapy for PCA, which alleviate pruritus and rashes without apparent adverse effects.

## Data Availability

The raw data supporting the conclusions of this article will be made available by the authors, without undue reservation.
